# Supplementary material for: Engineered autocrine signaling eliminates muscle cell FGF2 requirements for cultured meat production
Source: bioRxiv. 2023 Apr 17:2023.04.17.537163. Preprint. [Version 1] doi: 10.1101/2023.04.17.537163 (PMC10153192; doi:10.1101/2023.04.17.537163)
Supplement: Supplement 1 [file media-1.pdf]

Supplementary Materials: Engineered autocrine signaling eliminates muscle cell FGF2 requirements for cultured meat production

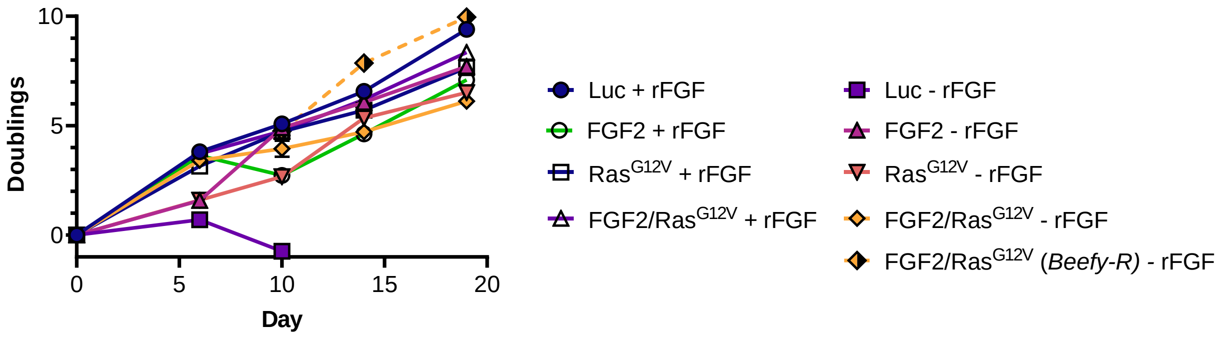

Figure S1: 19-day growth of all engineered cells +/- 40 ng/mL of exogenous recombinant FGF (rFGF). Results showed that growth is maintained for all cells except for luciferase control cells in the absence of exogenous rFGF (Luc – rFGF).

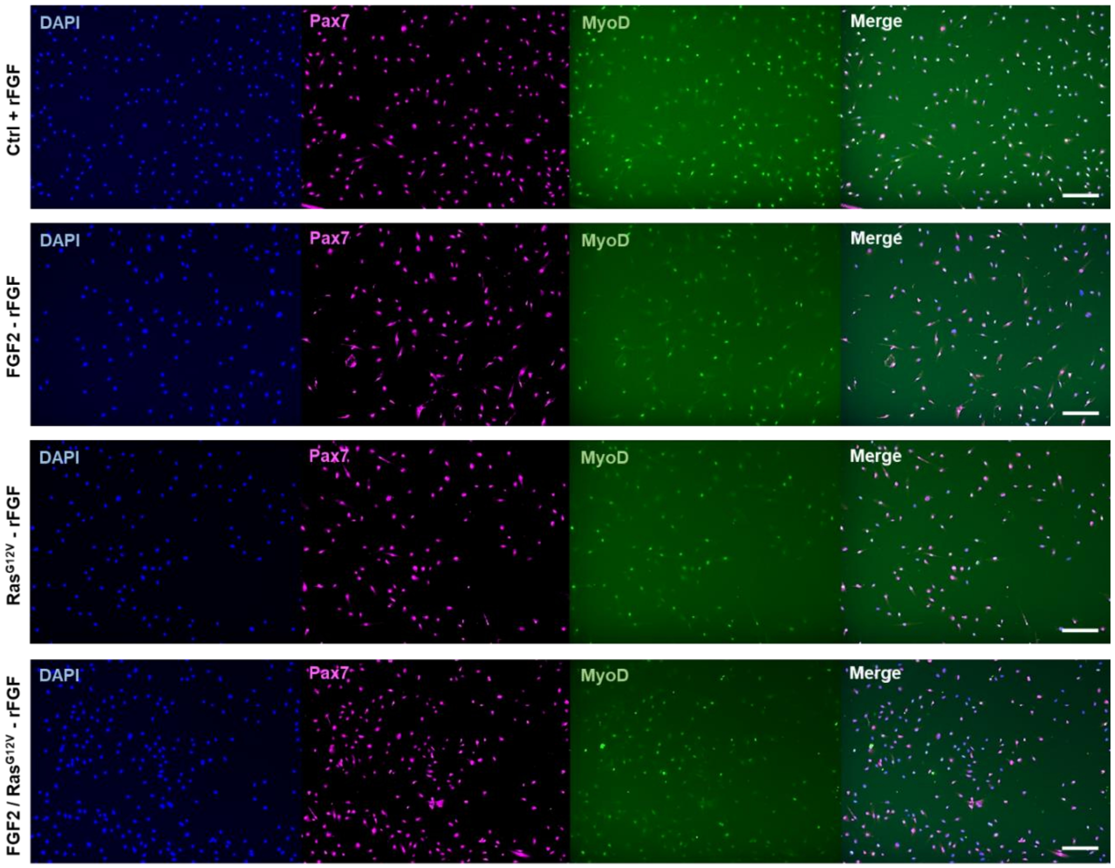

Figure S2: Immunostaining of proliferative cells for nuclei (DAPI), Paired-box 7 (Pax7), and Myoblast determination protein 1 (MyoD). Scale bars 200 µm.

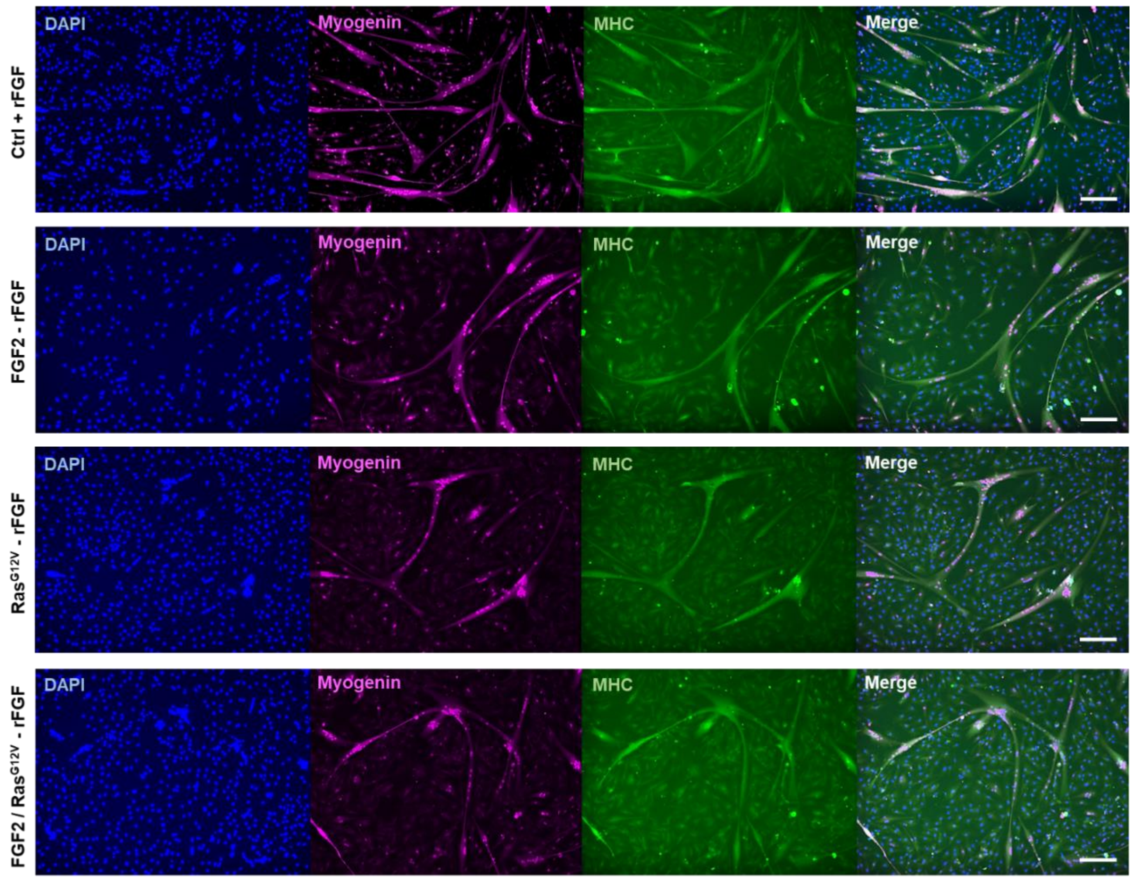

Figure S3: Immunostaining of differentiated cells for nuclei (DAPI), Myogenin, and Myosin Heavy Chain (MHC). Scale bars 200 µm.

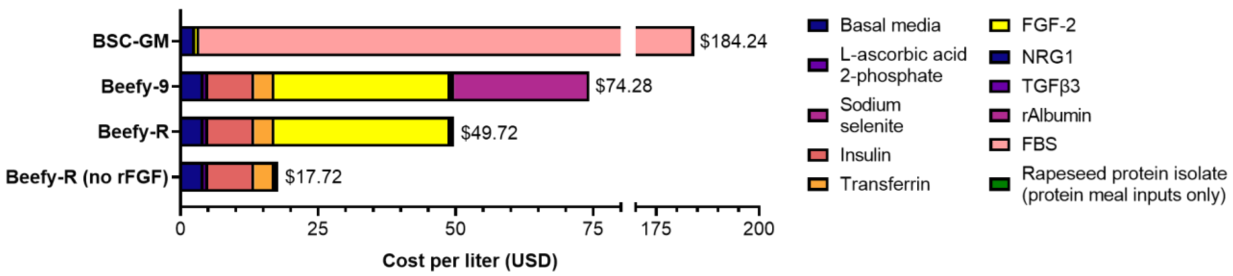

Figure S4: Cost analysis of serum-containing media (BSC-GM), serum-free Beefy-9 media, serum-free Beefy-R media (in which rAlbumin was replaced with rapeseed protein isolate), and Beefy-R without rFGF, which is made possible through autocrine engineering. Media prices based on bulk ordering of components described previously[1,2].

FGF2	ATGGCGGCAGGTTCAATAACTACCCTGCCTGCTCTGCCTGAGGATGGCGGATCTGGTGCTTTTCCACCTGG<br>CCACTTCAAGGACCCCAAGAGGCTGTACTGCAAGAACGGCGGATTCTTCCTGAGGATTCACCCCGACGGA<br>AGAGTGGATGGCGTGCGCGAAAAGAGCGACCCTCACATCAAGCTGCAACTGCAGGCCGAAGAGAGAGGC<br>GTCGTCAGCATCAAAGGCGTGTGCGCCAATAGATACCTGGCCATGAAGGAAGATGGCAGGCTGCTGGCCT<br>CCAAATGCGTGACCGACGAGTGCTTCTTCTTCGAACGCCTGGAAAGCAACAACTACAACACCTACAGAAG<br>CCGCAAGTACTCCTCTTGGTACGTGGCCCTGAAGAGGACCGGCCAGTACAAGCTGGGACCTAAGACAGGC<br>CCTGGACAGAAGGCCATCCTGTTCCTTCCAATGTCCGCCAAAAGTggatctggcgaaggcagaggctctctgctgacatgtggcga<br>cgtggaagagaaccctggacct
Ras^{G12V}	ATGACGGAATATAAGCTGGTGGTGGTGGGCGCCGTCGGTGTGGGCAAGAGTGCGCTGACCATCCAGCTGA<br>TCCAGAACCATTTTGTGGACGAATACGACCCCACTATAGAGGATTCCTACCGGAAGCAGGTGGTCATTGA<br>TGGGGAGACGTGCCTGTTGGACATCCTGGATACCGCCGGCCAGGAGGAGTACAGCGCCATGCGGGACCA<br>GTACATGCGCACCGGGGAGGGCTTCCTGTGTGTGTTTGCCATCAACAACACCAAGTCTTTTGAGGACATCC<br>ACCAGTACAGGGAGCAGATCAAACGGGTGAAGGACTCGGATGACGTGCCCATGGTGCTGGTGGGGAACA<br>AGTGTGACCTGGCTGCACGCACTGTGGAATCTCGGCAGGCTCAGGACCTCGCCCGAAGCTACGGCATCCC<br>CTACATCGAGACCTCGGCCAAGACCCGGCAGGGAGTGGAGGATGCCTTCTACACGTTGGTGCGTGAGATC<br>CGGCAGCACAAGCTGCGGAAGCTGAACCCTCCTGATGAGAGTGGCCCCGGCTGCATGAGCTGCAAGTGTG<br>TGCTCTCCTAA

Table S1: Genes used. Gene sequences used in constructs (upper case), followed by 2A linker sequences used after FGF-2 for bi-cistronic expression with Ras^{G12V} (lower case).

Component	Concentration	Supplier	Catalog #
**DMEM/F12 basal media**	**N/A**	**ThermoFisher**	**11320033**
**2-Phospho-L-ascorbic acid trisodium salt**	**200 µg/mL**	**Sigma**	**49752-10G**
**Insulin (human, recombinant)**	**20 µg/mL**	**Sigma**	**91077C-250MG**
**Transferrin (human, recombinant)**	**20 µg/mL**	**InVitria**	**777TRF029**
**Sodium selenite**	**20 ng/mL**	**Sigma**	**S5261-10G**
**Fibroblast growth factor (FGF-2)**	**40 ng/mL**	**PeproTech**	**100-18B**
**Neuregulin (NRG1)**	**0.1 ng/mL**	**PeproTech**	**100-03**
**Transforming growth factor (TGFβ3)**	**0.1 ng/mL**	**R&D Systems**	**8420-B3-005/CF**
**UltraPure Water**	**5.8% (v/v)**	**ThermoFisher**	**10977015**
**Antibiotic/Antimycotic**	**1% (v/v)**	**ThermoFisher**	**1540062**
**Recombinant albumin**	**0.8 mg/mL**	**Sigma**	**A9731-1G**

Table S2: Components of the various media used throughout this study. “Beefy-9 + FGF” had all components, “Beefy-9 – FGF” had all components except FGF-2, “B8 + FGF” had all components except recombinant albumin, and “B8 – FGF” had all components except FGF-2 and recombinant albumin.

References

1. Stout, A. J. *et al.* Simple and effective serum-free medium for sustained expansion of bovine satellite cells for cell cultured meat. *Commun Biol* **5**, 1–13 (2022).
2. Stout, A. J. *et al.* A Beefy-R culture medium: replacing albumin with rapeseed protein isolates. 2022.09.02.506409 Preprint at https://doi.org/10.1101/2022.09.02.506409 (2022).
